# Genetic and pharmacological regulation of the endocannabinoid CB1 receptor in Duchenne muscular dystrophy

**DOI:** 10.1038/s41467-018-06267-1

**Published:** 2018-09-27

**Authors:** Fabio A. Iannotti, Ester Pagano, Ombretta Guardiola, Simone Adinolfi, Valentina Saccone, Silvia Consalvi, Fabiana Piscitelli, Elisabetta Gazzerro, Giuseppe Busetto, Diego Carrella, Raffaele Capasso, Pier Lorenzo Puri, Gabriella Minchiotti, Vincenzo Di Marzo

**Affiliations:** 1 Endocannabinoid Research Group, Institute of Biomolecular Chemistry (ICB), National Research Council (CNR), 80078 Pozzuoli, Italy; 2 Department of Pharmacy, University of Naples Federico II, 80131 Naples, Italy; 3 Institute of Genetics and Biophysics, National Research Council (CNR), 80131 Naples, Italy; 4 IRCCS Fondazione Santa Lucia Rome, 00143 Rome, Italy; 5 Gaslini Children’s Hospital, L.go Gaslini 5, 16147 Genova, Italy; 6 Muscle Research Unit, Experimental and Clinical Research Center, Charit ´Universitätsmedizin and Max Delbrück Center, Berlin, 13092 Germany; 7 Department of Neuroscience Biomedicine and Movement, Section of Physiology and Psychology, University of Verona, 37134 Verona, Italy; 8 Telethon Institute of Genetics and Medicine (TIGEM), 80078 Pozzuoli, Italy; 9 Department of Agricultural Sciences, University of Naples Federico II, Portici, 80055 Italy; 10 Development, Aging and Regeneration Program, Sanford Burnham Prebys Medical Discovery Institute, 10901 N Torrey Pines Rd, La Jolla, CA 92037 USA; 11 Canada Excellence Research Chair, Institut Universitaire de Cardiologie et de Pneumologie de Québec and Institut sur la Nutrition et les Aliments Fonctionnels, Université Laval, Québec, QC, G1V 0A6 Canada

## Abstract

The endocannabinoid system refers to a widespread signaling system and its alteration is implicated in a growing number of human diseases. However, the potential role of endocannabinoids in skeletal muscle disorders remains unknown. Here we report the role of the endocannabinoid CB1 receptors in Duchenne’s muscular dystrophy. In murine and human models, CB1 transcripts show the highest degree of expression at disease onset, and then decline overtime. Similar changes are observed for PAX7, a key regulator of muscle stem cells. Bioinformatics and biochemical analysis reveal that PAX7 binds and upregulates the CB1 gene in dystrophic more than in healthy muscles. Rimonabant, an antagonist of CB1, promotes human satellite cell differentiation in vitro, increases the number of regenerated myofibers, and prevents locomotor impairment in dystrophic mice. In conclusion, our study uncovers a PAX7–CB1 cross talk potentially exacerbating DMD and highlights the role of CB1 receptors as target for potential therapies.

## Abstract (web summary)

The regenerative capacity of muscle stem cells is impaired in Duchenne muscular dystrophy (DMD). Here, the authors show that the endocannabinoid receptor CB1 is activated by PAX7 in muscle stem cells, and that pharmacological inhibition of CB1 promotes stem cell activation and ameliorates symptoms in DMD mouse models.

## Introduction

Duchenne’s muscle dystrophy (DMD) represents the most frequent form of hereditary myopathy. It is caused by mutations in the X-linked gene encoding for the structural protein dystrophin, which plays a critical structural role by being part of the dystrophin–glycoprotein complex that physically connects the cytoskeleton to the surrounding extracellular matrix through the cell membrane. Loss of dystrophin function caused by large intragenic deletions (65% of the cases), intragenic duplications (6–10% of the cases), or point mutations associated to other sequence variations (30–35% of the cases) leads to progressive and irreversible muscle wasting and weakness. Since the dystrophin gene is located on the X chromosome, the disease mostly affects young boys with a frequency of approximately 1:3500^1–5^.

Recent studies demonstrated that dystrophin plays a key role also in satellite cells, the muscle stem cells normally deputed to regenerate injured muscle fibers, where the lack of the functional protein causes asymmetric cell division, altered morphogenesis, and inefficient differentiation^6,7^. Although in both human and murine DMD skeletal muscles the number of satellite cells is higher than healthy tissue, their regenerative capacity is inevitably compromised along with disease progression^7–10^.

The endocannabinoid signaling system (ECS) is composed of a group of endogenous molecules including: (a) two endogenous lipid mediators, anandamide (AEA) and 2-arachidonoyl-glycerol (2-AG); (b) enzymes controlling AEA and 2-AG biosynthesis and degradation; and (c) two AEA and 2-AG responsive G protein-coupled receptors known as cannabinoid receptor of type 1 (CB1) and type 2 (CB2)^11–13^. In mammals, the function of the ECS is to control a large variety of physiological processes at both central and peripheral levels^11,12,14^. However, the potential role of the ECS in skeletal muscle disorders remains unknown. Recently, we have shown that the muscle levels of 2-AG are decreased during both myotube formation in vitro from C2C12 myoblasts and mouse muscle development in vivo. We also reported that in murine and primary human myoblasts the stimulation of CB1 by endogenous 2-AG or synthetic agonists such as arachidonoyl-2-chloroethylamide (ACEA), promotes myoblast proliferation while counteracting myoblast differentiation. Opposite effects were observed with rimonabant (SR141716) or AM251, two CB1 antagonists/inverse agonists^15^.

The aim of the present study was to explore the regulation and function of the endocannabinoid CB1 receptor in skeletal muscle, as well as isolated myoblasts and satellite cells from dystrophic mdx mice or human DMD patients. We demonstrate the existence of a functional interplay between CB1 and PAX7, a key factor regulating muscle regeneration through satellite cell division, and that antagonism of CB1 prevents the loss of muscle activity in dystrophic mice.

## Results

### CB1 and PAX7 expression in dystrophic skeletal muscles

In order to define the expression levels of CB1 in dystrophic muscles, we dissected from both control and mdx mice, the animal model more used to study DMD^16–18^, the quadriceps, diaphragm, soleus, and gastrocnemius muscles. All these tissues were isolated at three different time points: (1) before the onset of the disease (3 weeks of age); (2) at the onset of the disease (5 weeks), and (3) 3 weeks after the onset^19^.

**Fig. 1:**
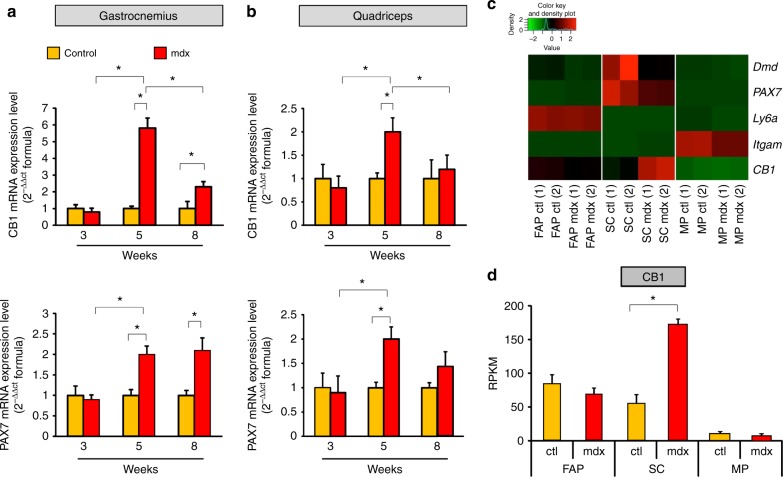
CB1 and PAX7 gene expression in dystrophic skeletal muscles and cells. **a, b** Time course of CB1 and PAX7 mRNA expression levels in gastrocnemius (**a**) and quadriceps (**b**) muscle of control (dark yellow columns) and mdx (red columns) mice of 3 (*n* = 8), 5 (*n* = 8), and 8 (*n* = 8) weeks of age. The quantification of transcripts for CB1 and PAX7 was performed by quantitative real-time PCR. **c** Heatmap representation of selected genes obtained from RNA-seq analysis in fibroadipogenic (FAP), satellite (SC), and macrophage (MP) cells isolated from 8-week-old control (*n* = 4) and mdx (*n* = 4) mice. Red, upregulated; green, downregulated. **d** Bar graph showing RPKM (Reads Per Kilobase of transcript per Million mapped reads) normalized values for the CB1 gene obtained from RNA-seq analysis in isolated FAP, SC, and MP cells. Each bar is the mean ± SEM of the independent determinations. *P ≤ 0.05 vs. control animals or cells, determined by Student’s *t* test

Our qPCR analysis revealed that in the gastrocnemius and quadriceps (vastus intermedius), CB1 mRNA levels show a bell-shaped profile with the highest degree of expression at disease onset and declining then over time (Fig. 1a, b, upper panel). In control muscles, instead, the mRNA levels of CB1 were always comparable to the levels observed at 3 weeks in mdx mice (Fig. 1a, b, upper panel). Intriguingly, the transcript levels of PAX7, the most known master gene regulating satellite cell activation and self-renewal^20,21^, showed an expression profile very similar to CB1 (Fig. 1a, b, lower panel). A similar expression profile of CB1 and PAX7 was also found in the diaphragm and soleus muscle (data not included). To gain a mechanistic understanding relative to the upregulated expression of CB1 gene in DMD muscles, and to define the specific muscle cell subpopulation responsible for these changes, we performed RNA-sequencing (RNA-seq) experiments on fluorescence-activated cell sorting (FACS)-sorted satellite, macrophage and fibroadipogenic progenitor (FAP) cells freshly isolated from the hind limb muscles of 8-week-old control and mdx mice. As shown in the heat map in Fig. 1c, this approach revealed that CB1 is expressed in satellite, FAP, and, much less, macrophage cells. The expression of specific markers of these cell subtypes is also shown (Fig. 1c). Most importantly, we demonstrated that the lack of dystrophin was accompanied by a significant increase in the transcript levels of CB1, occurring exclusively in satellite cells but not in FAP or macrophages (Fig. 1c). The graph bar (Fig. 1d) shows the quantification of the results. Instead, CB2 was only weakly expressed in satellite or FAP cells, and abundantly present in macrophages, where its expression was significantly increased in mdx mice (Supplementary Figure 1).

To clarify whether the increased expression of CB1 observed in dystrophic muscles was due to increased mRNA expression per cell or instead depended on the increased number of PAX7^+^ cells, we isolated satellite cells from the gastrocnemius of control and mdx mice during (5 weeks) and after (8 weeks) the onset of pathology. Our results, in agreement with previous findings^6–8^, indicate that the total number of satellite cells isolated from skeletal muscles of mdx mice was significantly higher than in control mice at both time points (Supplementary Figure 2). However, the fold-increase in satellite cell number appeared to account for increased expression of CB1 and PAX7 only at week 8 (approximately twofold increase of cell number), and much less at week 5 (~1.5-fold of cell number), when the latter and, particularly, the former gene appeared to be expressed more-fold (approximately fivefold and twofold) than what could be accounted for by the increase of satellite cells.

### CB1 receptor expression profile in human DMD muscles

**Fig. 2:**
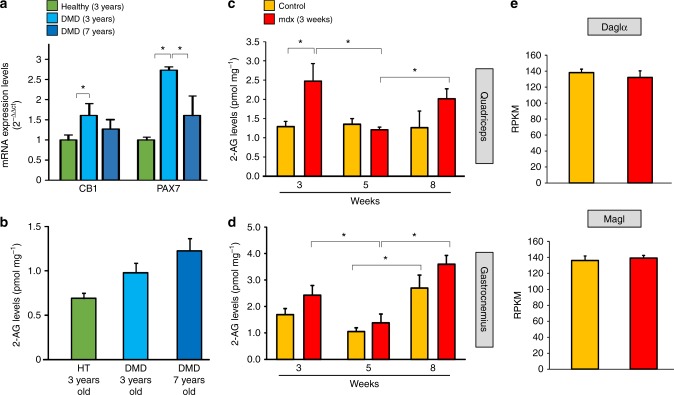
CB1 and PAX7 expression and 2-AG levels in dystrophic muscles. **a** mRNA expression levels of CB1 and PAX7 in muscle samples of healthy children (HT, controls *n* = 3) or DMD donors of 3 (*n* = 4) and 7 (*n* = 4) years. **b** Measurement of the endogenous levels of 2-AG in muscle samples of the same healthy and DMD donors. Time course of the endogenous levels of 2-AG in the quadriceps (**c**) and gastrocnemius (**d**) muscle of control (dark yellow; *n* = 8) and mdx (red columns; *n* = 8) mice. The levels of 2-AG are expressed as pmol mg^−1^ of wet tissue weight. *P ≤ 0.05 vs. control group, determined by Student’s *t* test. **e** The bar graphs show RPKM (Reads Per Kilobase of transcript per Million mapped reads) normalized values for the Daglα and Magl genes obtained from RNA-seq analysis in satellite cells isolated from 8-week-old wt (*n* = 4) and mdx mice (*n* = 4)

Since mdx mice exhibit etiological and symptom differences with human DMD^17,18,22^, we evaluated the expression profile of CB1 and PAX7 genes in leg muscle biopsy specimens from both healthy and DMD human donors. As shown in Fig. 2a, both genes were significantly upregulated in the muscle of 3-year-old boys affected by DMD, when compared to age-matched healthy controls, and lower in 7-year-old compared to 3-year-old DMD boys. Data from healthy 7-year-old patients are not available, since all the collected samples are usually true positives at this age due to the fact that boys affected by DMD usually exhibit the first symptoms around 3–5 years of age ^23^.

### 2-AG levels in dystrophic murine and human muscles

In both whole muscles and myoblasts of DMD boys (Fig. 2b and Supplementary Table 1) as well as in the muscles of mdx mice (Fig. 2c, d), we found levels of 2-AG in the µM range sufficient to constitutively activate CB1 receptors. No statistically significant differences were observed in 2-AG levels of healthy and DMD donors (Fig. 2b), while higher levels of 2-AG were found in the muscles of 3-week-old mdx mice (Fig. 2c, d). However, 2-AG levels in both mdx mouse quadriceps and gastrocnemius, and in control gastrocnemius, first decreased (from 3 to 5 weeks of age) and then increased (from 5 to 8 weeks of age). Interestingly, 2-AG levels in the gastrocnemius were higher in mdx mice at 8 weeks than 3 and 5 weeks, when the tissue contained also the highest number of satellite cells (Supplementary Figure 2), in agreement with the hypothesis that these cells are an important source of the endocannabinoid. Satellite cells from control and mdx mouse hind limb muscles expressed similar levels of mRNAs of enzymes for 2-AG biosynthesis and hydrolytic inactivation (Fig. 2e), confirming that these cells contain the enzymatic apparatus to produce and inactivate 2-AG.

### PAX7 directly regulates the transcriptional activity of CB1 gene

**Fig. 3:**
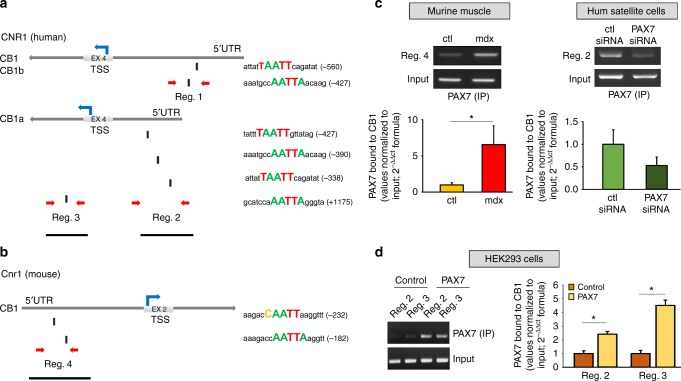
Bioinformatics and ChIP analysis. Schematic representation of the human (**a**) or murine (**b**) CB1 gene. The transcriptional start site (TSS) for each gene isoform is indicated. The identified PAX7 sites are shown as small black rectangles below the schematic. Regions identified as 1–4, indicated by red arrows, correspond to PAX7-containing gene regions of high sequence homology in human and murine CB1 gene. **c** PAX7 occupancy of identified sites in the CB1 gene evaluated by chromatin immunoprecipitation (ChIP) analysis. Left: average data of the relative amount of the PAX7-immunoprecipitated DNA in the murine quadriceps muscle isolated from either wild-type or mdx mice; Right: average data of the relative amount of the PAX7-immunoprecipitated DNA in PAX7-silenced satellite cells. Data are from six separate experiments and normalized relative to the input DNA. The inset shows a representative agarose gel electrophoresis of the qPCR products obtained from PAX7-immunoprecipitated DNA for each experimental condition. **d** Left: representative agarose gel electrophoresis of the qPCR products obtained from PAX7-immunoprecipitated DNA for each experimental condition. Right: average data of the relative amount of the PAX7-immunoprecipitated DNA in HEK293 cells transfected with control scramble (*n* = 4) human PAX7 construct (*n* = 4). *P ≤ 0.05 vs. control group, determined by Student’s *t* test

In view of the very similar expression profile of PAX7 and CB1 in both human and murine skeletal muscles affected by DMD, we explored the possibility of a direct regulation of CB1 gene transcription by PAX7. To prove this, we used bioinformatics tools to search for putative consensus sequences for PAX7 within the CB1 gene. The human CB1 gene (CNR1) produces six splice variants. Transcript variants 1, 3, 4, 5, and 6 encode full-length CB1, which is 472 amino acids in length encoded without interruption by a single region in exon 4. Exon 4, however, can be differentially spliced to remove 102 nucleotides (nts). This splicing occurs in transcript variant 2 that encodes the truncated CB1 isoform b. The third isoform, named CB1a (or CB1 short), differs by 167 nts from the isoform b. Furthermore, two transcriptional start sites (TSS) are present at the 5′ end of exon 4. Translation from the former site produces CB1 and CB1b. Translation from the latter site is thought to produce the amino-terminal variant CB1a^24^. On the other hand, the mouse and rat CB1 genes are located on chromosomes 4 and 5, respectively. A single coding variant of CB1 has been identified in these two species^25^. We identified distinct putative PAX7 binding sequences with a high homology score (>90) in both human and murine CB1 encoding genes. In human CB1 gene, we found two PAX7-rich sites located at −560 and −427 bp from the TSS producing the isoforms CB1 and CB1b. Three rich regions were instead found at −427, −390, and −338 from the TSS producing the isoform CB1a or CB1 short. Additionally, a further PAX7-rich region was detected 1175 bp downstream the TSS of CB1a (Fig. 3a). In murine CB1 gene, we found two distinct putative consensus regions for PAX7 located in the 5′UTR region at −232 and −182 bp of distance from TSS (Fig. 3b). Chromatin immunoprecipitation (ChIP) analysis showed that the transcription factor PAX7 effectively binds the putative responsive sequences identified within the CB1 encoding genes. Briefly, in these experiments, the genomic DNA was isolated from gastrocnemius muscles of both control and mdx mice at 5–6 weeks of age and used for the immune-affinity reaction with a specific anti-PAX7 antibody. Subsequently, using specific pair of primers able to amplify the genomic portion of interest of the CB1 gene containing the putative PAX7 binding sites (Fig. 3a, b, red arrows), we found that PAX7 effectively binds these sites and that the amounts of the complex are significantly higher in muscles of dystrophic than healthy mice (Fig. 3c, left). To prove the binding of PAX7 to the human CB1 gene, we repeated the ChIP procedure in primary human PAX7-silenced satellite cells. In these latter experiments, due to the fact that ChIP analysis requires a large number of cells, we were able to study the potential binding of PAX7 to CB1 only within region 2. This portion of the gene was preferred to the other two (regions 1 and 3) because it contains the highest number of putative PAX7 binding sites (Fig. 3a). Furthermore, we transfected human satellite cells with siRNA sequences against PAX7 and collected them after 48 h to perform ChIP analysis. As shown in the right panel of Fig. 3c (right), we found in PAX7-silenced primary satellite cells a ~50% reduction of the intensity of the signal corresponding to the binding of PAX7 to CB1.

To test the efficacy of the siRNA sequences used to target PAX7, we performed qPCR and western blot analyses, which confirmed not only the knockdown of the transcription factor but also that of its major target LIX1^26^, and of the CB1 (but not CB2) gene (Supplementary Figure 3).

**Fig. 4:**
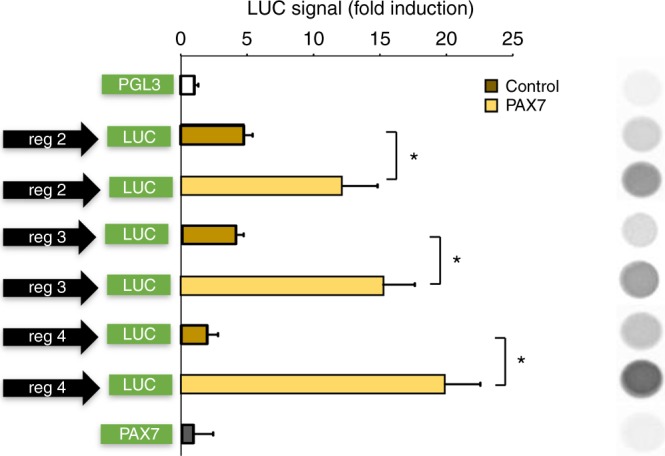
Luciferase assay in PAX7-transfected HEK293 cells. Relative luciferase activity following transfection of human constructs into HEK293 cells. Cells were co-transfected with the pGL3 promoter vector carrying the construct of interest (regions 2, 3, and 4) cloned upstream of the luciferase gene together with an equimolar amount of plasmid encoding for human PAX7. For the control condition, PAX7 plasmid was replaced with pCDNA3.1. Cells were co-transfected with a β-Gal encoding vector to normalize transfection efficiency and the signal intensity of luciferase. The inset on the right shows a representative chemiluminescent signal emitted from the reaction of luciferase from the different experimental conditions. Data are expressed as the mean ± SEM of four independent determinations. *P ≤ 0.05 vs. control group, determined by Student’s *t* test

Finally, we utilized human embryonic kidney (HEK293) cells to overcome the problems due to the difficulty of isolating a sufficient number of human satellite cells to perform functional luciferase assays. When HEK293 cells were transiently transfected with human PAX7 or control plasmid (scramble), our ChIP analysis confirmed that PAX7 binds with high affinity the endogenous CB1 gene within regions 2 and 3 (Fig. 3d). We then confirmed that PAX7 binding to CB1 enhances its transcription by performing luciferase assays following the cloning of the portion of either the human or murine CB1 gene containing the PAX7 consensus regions in a PGL3 plasmid, upstream of the luciferase gene. In particular, we cloned: a) two human CB1 genomic portions of interest including the two distinct PAX7 consensus regions identified at 5′UTR and downstream the TSS region of CB1a (370 and 319 bp, black bars Fig. 3a, b) the murine CB1 portion of interest, including the two sites identified in the 5′UTR region (703 bp, black bar Fig. 3b). The three constructs were separately transfected into HEK293 cells with or without a plasmid expressing PAX7, which expressed the protein at detectable levels in a variety of non-muscle cell lines. As shown in Fig. 4, when HEK293 cells were transfected with the plasmid carrying the murine or human CB1 genomic portion of interest in the presence of PAX7, the chemiluminescent signal intensity was significantly higher (by about threefold and 6.5-fold, respectively) than when PAX7 was not co-expressed.

Taken together, these results indicate that the transcriptional factor PAX7 directly regulates CB1 gene expression by promoting its transcriptional activity in both murine and human species, showing more efficacy during DMD progression.

### Pharmacological role of CB1 in human muscle cells

**Fig. 5:**
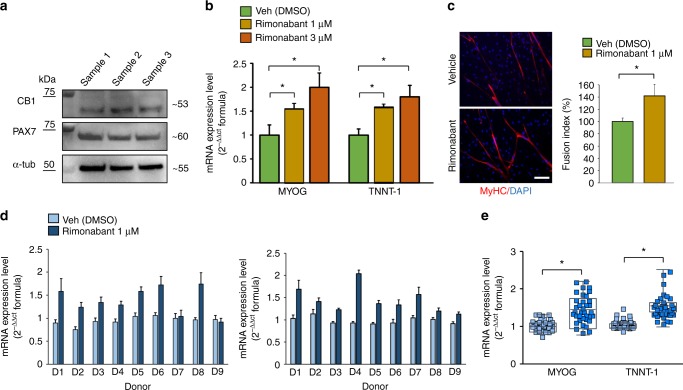
Pharmacological role of CB1 in primary human muscle cells. **a** Representative blot for CB1 and PAX7 protein expression in primary human satellite cells. The approximate molecular mass for each of these proteins (expressed in kDa) is shown on the right. **b** Bar graph showing the mRNA expression levels of MYOG and TNNT-1 in satellite cells exposed to DM for 5 days +/− rimonabant 1–3 µM. Each bar is the mean ± SEM of four separate determinations. **c** Morphological analysis of myotube formation in human primary satellite cells exposed to DM for 5 days in the presence of vehicle (DMSO, control) or rimonabant 1 µM. MyHC (red) and DAPI (blue). (Scale bar, 10 μm). The fusion index was calculated in both vehicle (DMSO)- and rimonabant-treated cells exposed to DM for 5 days. The asterisk denotes the *P* ≤ 0.05 vs. vehicle-treated cells. **d** Bar graphs showing the MYOG and TNNT-1 mRNA expression levels in primary human myoblasts isolated from each DMD donors (D1–D9) and induced to differentiate in presence of vehicle (DMSO) or rimonabant (1 µM). The quantification of transcripts was performed in quadruplicate by quantitative real-time PCR. The error bars correspond to the internal SEM. **e** The graph shows the differences in the expression levels of MYOG and TNNT-1 between vehicle- or rimonabant-treated myoblasts calculated by combining the DMD patient’s results together. Each bar is the mean ± SEM of the data from the nine patients, each of which was obtained from at least four separate determinations (see **d**). *P ≤ 0.05 vs. vehicle group, determined by Student’s *t* test

Using western blot analysis, we found that the CB1 protein is expressed in PAX7-positive primary human satellite cells (Fig. 5a). Then, when these cells were induced to differentiate into myotubes, we found that blockade of CB1 receptor by rimonabant (1–3 µM) increased the expression of troponin-T-1 (TNNT1), myogenin (MYOG), and myosin heavy chain (MyHC), canonical markers of myogenesis^27^ (Fig. 5b, c), while it reduced the proliferation rate of satellite cells (Supplementary Figure 4). The stimulation of CB1 by 2-AG or ACEA (3 µM) produced the opposite effects (Supplementary Figure 4).

To test a potential endocannabinoid-based therapeutic treatment for DMD patients, and given the remarkable difficulty in isolating satellite cells from a muscle biopsy specimens^28^, we isolated primary myoblasts from nine different donors (ranging from 1 to 7 years old) diagnosed with DMD caused by different mutations in the dystrophin gene (Supplementary Table 2). Proliferating myoblasts at a confluency of about 80–90% were exposed to the differentiation media plus vehicle (dimethyl sulfoxide (DMSO)) or rimonabant (1 µM) for 5 days. At the end of treatment, the expression of MYOG and TNNT-1 was quantified by qPCR analysis. We found that rimonabant increased the expression of TNNT-1 and MYOG in differentiating myoblasts isolated from patients D1 to D6, whereas in patients D7-D8 and in patient D9 there was a partial or no effect, respectively (Fig. 5d). However, when combining the results of myoblasts from all patients, we observed a significant difference in the expression levels of MYOG and TNNT-1 between vehicle (DMSO) and rimonabant-treated myoblasts (Fig. 5e).

### Effect of CB1 antagonism on locomotor activity in mdx mice

**Fig. 6:**
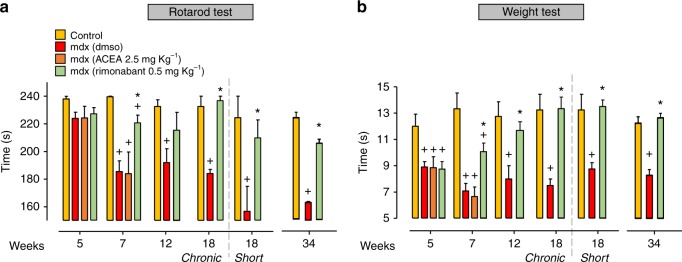
Effect of rimonabant on locomotor activity of mdx mice treated. The muscle coordination and strength was measured in control (*n* = 7) and mdx mice treated with vehicle (DMSO, *n* = 11), ACEA (2.5 mg Kg^−1^, *n* = 7), or rimonabant (0.5 mg Kg^−1^, *n* = 16 IP every other day) at the beginning of the treatment (5 weeks) and at 7, 12, and 18 (chronic) weeks. The treatment was repeated starting from week 16 until week 18 of age (18 *short*, control wt = 7; mdx + DMSO = 10; mdx + rimonabant = 10) or from week 32 until week 34 of age (control wt = 7; mdx + DMSO = 10; mdx + rimonabant = 10) by the rotarod (**a**) and weight (**b**) tests. Each bar is the mean ± SEM. ^+^*P* ≤ 0.05 vs. control group, *P ≤ 0.05 vs. mdx vehicle group, determined by Student’s *t* test

We next treated mdx mice with ACEA or rimonabant and measured their muscle coordination and strength by the use of rotarod and weight holding tests. These are currently the most commonly used tests to evaluate muscle skills in dystrophic mice^29,30^. At the week of disease onset (5 weeks), dystrophic mice were randomized into three groups, receiving vehicle (DMSO, control), 0.5 mg Kg^−1^ rimonabant, or 2.5 mg Kg^−1^ ACEA. Each drug was injected intraperitoneally three times per week. As shown in Fig. 6a, at 5 weeks, the locomotor activity of mdx mice, when compared to control animals, was significantly reduced, and then further worsened at 7 weeks (Fig. 6a). At 7 weeks, mdx mice treated with rimonabant, but not ACEA, for 2 weeks showed a significant recovery of their muscle coordination (Fig. 6a). In order to gain information about the effect of rimonabant in a more advanced stage of disease, we repeated the rotarod test at 12 and 18 weeks in the same group of mice chronically treated with rimonabant every other day commencing from week 5 (Fig. 6a, the treatment is indicated as chronic). In addition, we also looked at control and mdx mice at an even later stage following rimonabant treatment given every other day starting from week 16 until week 18 (Fig. 6a, the treatment is indicated as 18 short). In either case, rimonabant still prevented the loss of motor coordination. In the weight test, mdx mice were held by the middle/base of their tail to allow them to grasp the weight (from 20 to 60 g). From the moment the mouse grasps the weight with its forepaws, the interval of time during which the mouse can hold the weight is recorded and a score is assigned^30^. Also in this case, we found that, as compared to control, in mdx mice muscle strength was significantly impaired at 5 weeks of age, and that, at 7 weeks, rimonabant, but not ACEA, significantly rescued the loss of strength (Fig. 6b). The rescue was also observed at 12 and 18 weeks following both treatment regimens with rimonabant (Fig. 6b). Finally, the above therapeutic effects of rimonabant were confirmed also with a 2-week late treatment in aged mice, from 32 to 34 weeks (Fig. 6a, b).

### Effect of CB1 antagonism on muscle regeneration in mdx mice

**Fig. 7:**
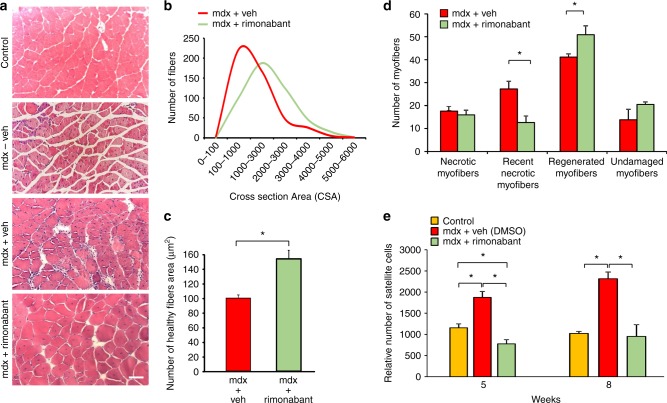
Effects of rimonabant on muscle degeneration. **a** Representative photomicrographs of H&E-stained transverse sections of gastrocnemius muscles isolated from wild-type (control, *n* = 6); mdx mice (without vehicle, *n* = 6); mdx mice treated with vehicle (DMSO, *n* = 6), or rimonabant (*n* = 6) 0.5 mg Kg^−1^ IP from week 5 to week 7 of age. Scale bars = 100 μm. **b** Total number of myofibers within each cross-sectional area (CSA, µm^2^). **c** number of healthy myofibers within each cross-sectional area (CSA, µm^2^). **d** Bar graph indicates the number of necrotic, partially regenerated (recently necrotic), fully regenerated and undamaged myofibers in control (DMSO) and rimonabant-treated mdx mice. Mice were treated from week 5 to week 7 of age. Each bar is the mean ± SEM. *P ≤ 0.05 vs. vehicle group, determined by Student’s *t* test. **e** Bar graph showing the relative number of satellite cells found in the gastrocnemius muscles of control and mdx mice treated with vehicle (DMSO) or rimonabant. Each bar is the mean ± SEM of four independent determinations

We next isolated skeletal muscles from mdx mice at the end of treatment to perform histological analyses following sectioning and staining with hematoxylin and eosin (H&E), which is commonly used to evaluate changes in the number of healthy or necrotic myofibers, position of nuclei within each myofiber, tissue infiltration of inflammatory or fat cells, fibrosis, etc^31^. The H&E staining revealed that in the gastrocnemius of 7-week-old dystrophic mice, there was a significant degeneration of skeletal muscle structures as compared to control animals of the same age, as shown in representative images (Fig. 7a). This was characterized by a robust reduction in the number of myofibers or of their diameter with centrally positioned nuclei (Fig. 7a). Treatment with rimonabant prevented these features (Fig. 7a) and significantly increased the number of regenerated myofibers, which exhibited larger size than both wild type and control group (Fig. 7a). All the effects observed in rimonabant-treated mdx mice were quantified and reported in the graphs (Fig. 7b–e). Somehow less strong, but still significant, protective effects were found in the quadriceps (vastus intermedius) (Supplementary Figure 5). This different efficacy might be due to the higher proportion of slow twitch fibers in vastus intermedius than in gastrocnemius, where CB1 is more abundantly expressed^32,33^ (Supplementary Figure 5). This could suggest that tonic activation of CB1 in the quadriceps is lower than in the gastrocnemius, also by virtue of the lower levels of 2-AG between 5 and 8 weeks of age (Fig. 2), and hence less efficaciously unmasked by a saturating dose of rimonabant. In addition, we isolated satellite cells from the gastrocnemius of control and dystrophic mice treated with rimonabant or vehicle. We found that, in mdx mice, rimonabant significantly reduced the number of satellite cells as compared to vehicle-treated mice and, at week 5, also to healthy mouse gastrocnemius (Fig. 7e), in agreement with the pro-differentiating effect of the CB1 antagonist observed here also in vivo (see below).

In summary, we show that in vivo, more prominently than in vitro, CB1 antagonism reduces the amount of proliferating satellite cells. In addition, the fact that after treatment with rimonabant the number of proliferating satellite cells was lower than in control mouse muscles, likely indicates that in dystrophic muscles PAX7 positively regulates CB1 to promote its tonic activation by endocannabinoids, which in turn increases the proliferative status of satellite cells.

### Rimonabant reduces the expression of degenerative markers

**Fig. 8:**
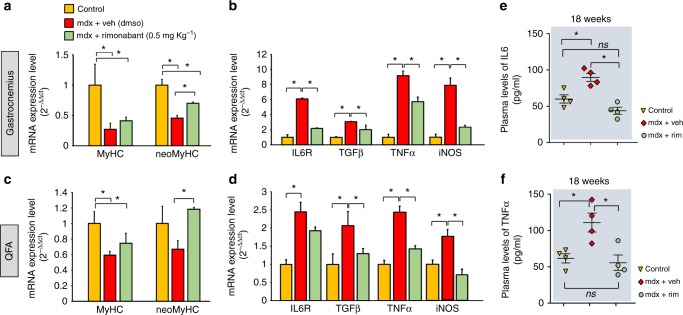
Effect of rimonabant on the expression of inflammation markers. The bar graph shows the expression levels of myosin heavy chain (MyHC) and neonatal-myosin heavy chain (neo-MyHC) (**a** and **c**) and/or transforming growth factor β 1 (TGF-β1), tumor necrosis factor α (TNFα), interleukin receptor 6 (IL6R), and inducible nitric oxide synthase (iNOS) (**b** and **d**) in the indicated skeletal muscles isolated from control (dark yellow), mdx mice + vehicle (DMSO, red columns) and mdx mice + rimonabant (0.5 mg Kg^−1^, green columns). Each bar is the mean ± SEM of six separate determinations. **e**, **f** Plasma levels of IL6 and TNFα quantified by ELISA in control (*n* = 4) and mdx mice treated with vehicle (DMSO; *n* = 4) or rimonabant 0.5 (*n* = 4) mg Kg^−1^. *P ≤ 0.05 vs. vehicle group, determined by Student’s *t* test

We evaluated the transcript levels of known genes involved in the inflammatory and degenerative process in the muscles of control and mdx mice of 7 weeks treated with rimonabant or vehicle. In the gastrocnemius and quadriceps of dystrophic mice, compared to control littermates, we found a significant decrease in the expression level of MyHC and neoMyHC (Fig. 8a, c, e), and, conversely, an increase of the inflammatory markers, interleukin 6 receptor (IL6R), tumor necrosis factor-α (TNFα), transforming growth factor β (TGF-β), and inducible nitric oxide synthase (iNOS) (Fig. 8a, d, f). In these muscles, 2-week treatment with rimonabant caused a partial or full recovery in the expression levels of neoMyHC and MyHC (Fig. 8a, c, e), whilst reducing the transcript levels of IL6R, TNFα, TGF-β, and iNOS (Fig. 8b, d, f). We also measured by ELISA the plasma levels of IL6 and TNFα in control and mdx mice treated with rimonabant from 5 to 18 weeks of age every other day. As expected, plasma levels of both IL6 and TNFα were considerably increased in mdx mice, and rimonabant fully reversed this effect (Fig. 8e, f).

### Intracellular mechanism coupled to CB1 receptor stimulation

**Fig. 9:**
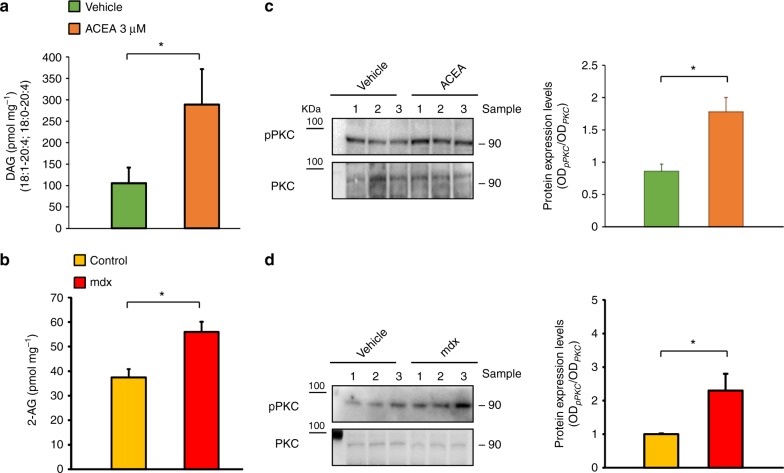
Measurement DAGs levels and activity in satellite cells and muscles. Levels of diacylglycerols (DAGs) (**a**) and 2-AG (**b**), in primary satellite cells exposed to the selective CB1 agonist ACEA 3 µM for 20 min. The levels of 2-AG and DAGs are normalized to the amount of lipids extract (pmol mg^−1^). Each bar is the mean ± SEM of six separate determinations. *P ≤ 0.05 vs. vehicle group, determined by Student’s *t* test. **c** Representative blots showing the chemiluminescent signal generated by the anti-phosphospecific (Thr497 site) and non-phosphospecific PKC antibody in satellite cells exposed to ACEA 3 µM for 20 min or **d** in the gastrocnemius isolated from control (*n* = 4) and mdx (*n* = 4) mice at 8 weeks. Quantification of phospho-PKC (pPKC) levels are normalized to total PKC. Each bar is the mean ± S.E.M. of five separate determinations. *P ≤ 0.05 vs. the indicated experimental group, determined by Student’s *t* test

In our previous study, we demonstrated that the stimulation of CB1 activates a typical G protein-coupled receptor-mediated signaling mechanism, that is, the hydrolysis of 4,5-bisphosphate (PIP2), thereby causing the inhibition of myogenesis-promoting voltage-gated potassium K_v_7.4 channels^12^. The formation of diacylglycerols (DAGs) and inositol trisphosphate (IP3) is known to occur as consequence of protein Gq-mediated PIP2 hydrolysis. Therefore, in order to characterize also in satellite cells the CB1 signaling cascade, we measured the levels of DAGs following CB1 stimulation with ACEA. We found that exposure of satellite cells to ACEA (3 µM) for 20 min elevated the intracellular levels of two arachidonic acid-containing DAG species. Since these DAGs may also act as biosynthetic precursors of 2-AG^34^, we also measured the levels of this endocannabinoid, and found them to be significantly increased (Fig. 9a, b). These results raise the possibility that CB1 stimulation may generate a positive loop through increased production and signaling of 2-AG.

In addition, being the members of protein kinase C subfamily (PKCs) preferential targets of DAGs in different cell types^35,36^, we hypothesized their potential involvement as downstream targets of CB1 receptors. It is known that PKC activation occurs through phosphorylation at distinct sites including Thr-494, Thr-495, and Thr-497^36^. Therefore, by means of western blot analysis performed using a selective phospho-specific antibody directed against the Thr497 site (common to all PKC isoforms), we analyzed potential changes in PKC phosphorylation in human satellite cells following CB1 stimulation. We found that, in agreement with its stimulation of DAG production, ACEA also increased phosphorylation of PKCs at Thr497, without affecting their expression (Fig. 8c). Notably, in the gastrocnemius of mdx mice at 8 weeks, we found a significant increase in PKC phosphorylation, which might depend on the tonic activation of CB1 by 2-AG, when the levels of this endocannabinoid are highest (Fig. 8d). It is worth noting that PKCs are known to: (a) directly inhibit voltage gated K_v_7 K^+^ channel activity^37^ and (b) exacerbate the inflammatory and immune response in DMD^38,39^. These results further support the potential use of CB1 antagonists, rather than agonists, to treat DMD.

## Discussion

Although great advances have been made toward a long-term therapeutic approach to treat DMD, there is still no cure. Complementary and/or supportive therapies are currently used to delay and/or reduce the severity of symptoms^2^. Our previous finding of the crucial role of the endocannabinoid 2-AG and its receptor, CB1, in controlling myoblast proliferation and differentiation in vitro and during early muscle development^12^ raised two important questions: (1) is the endocannabinoid system important also for the very first step in muscle formation, i.e., the generation of myoblasts from satellite cells? (2) Can pharmacological manipulation of CB1 receptors be useful to help treating the symptoms, or delaying the progress, of muscular dystrophies such as DMD? The former question is also relevant to the latter one, since satellite cell dysfunction represents a major feature in muscles affected by DMD. In fact, it was reported that the number of these cells is higher in DMD muscles^6–9^ (a finding confirmed in the present study), although their ability to form mature myotubes is compromised, this being one of the main causes of the defective skeletal muscle repair observed in dystrophic muscles^40,41^; whereas satellite cell number remains unchanged in less severe forms of muscular dystrophy^42^. Evidence indicates that satellite cell functionality is also indirectly affected by the hostile, e.g., pro-inflammatory and pro-fibrotic, microenvironment of the dystrophic muscle^40,43^, which antagonism of CB1 should also ameliorate^44^.

Here we showed that: (1) CB1 is overexpressed in the skeletal muscle and satellite cells of mdx mice, as well as in muscle biopsies of DMD patients, when the first signs of the disease occur, and concomitantly with a peak in expression of PAX7, a key transcription factor and biomarker for satellite cells; (2) CB1 upregulation in satellite cells of mdx mice cannot be accounted for uniquely by the increased number of these cells in dystrophic muscles, especially at the onset of disease, and hence must be the result of the overactivation of some regulatory pathway; (3) accordingly, PAX7 upregulates the expression of both human and mouse recombinant CB1 in host cells, and binds to the *Cnr1* gene in wild type and, to a higher extent, mdx mouse muscle, using specific consensus sequences on this gene; (4) antagonism of CB1 receptors, opposite to their activation, reduces human satellite cell proliferation; in addition, it enhances the formation of myotubes from either satellite cells from healthy tissue, or human myoblasts from DMD patients; and (5) CB1 antagonism in mdx mice, starting at an early age, when their skeletal muscle exhibit the highest expression levels of CB1 and sufficient amounts of 2-AG to activate them, might represent a tool to counteract the loss of muscle strength in dystrophic mice, by enhancing muscle tissue regeneration and possibly reducing inflammation and fibrosis.

In the muscle tissues of young boys with DMD, we found low micromolar concentrations of 2-AG and high CB1 expression. This observation led us to hypothesize that increased endocannabinoid tone in dystrophic muscles could somehow participate in the reduced ability of muscle precursor cells to terminate their differentiation into mature myotubes. This hypothesis was confirmed by our findings in: (1) primary human satellite and/or myoblast cells isolated from healthy and DMD patients, where antagonism of CB1 receptor promoted differentiation; and (2) mdx mice, where rimonabant increased significantly the number of healthy/regenerating fibers, and decreased inflammatory markers. It is not possible to assess whether the effect of rimonabant on satellite cell differentiation was due to reduced proliferation or vice versa. Since CB1 activation by ACEA significantly enhanced proliferation but appeared to reduce differentiation to a smaller extent, it is possible that the two processes are not solely the consequence of the other, and, in this case, CB1 antagonists would have the advantage of independently ameliorating two signs of DMD, i.e., satellite cell excessive proliferation and impaired differentiation. Likewise, from our biochemical and histological analyses of mdx mice at the end of treatment, it is not possible to conclude whether the effect of rimonabant in vivo on muscle regeneration is the product of reduced inflammation or if the latter effect is a consequence of improved muscle regeneration. Appropriate studies, with time-course analyses of the various effects of rimonabant, need to be performed in order to answer this question, and might demonstrate two independent effects of CB1 antagonism, as suggested by our present data in human DMD myoblasts and previous evidence of the anti-inflammatory properties of rimonabant^45,46^. Interestingly, the antagonist did not enhance to the same extent differentiation of myoblasts from DMD patients with different mutations of the dystrophin gene. There is clinical evidence demonstrating that the type of mutated dystrophin does not necessarily correlate with the severity of the disease^47,48^. Often, in patients with the same mutation disease severity can vary, sometimes even within the same family^49,50^. Therefore, it is difficult to correlate the different pro-differentiating effects of rimonabant in myoblasts isolated from different patients with the potential clinical or genetic characteristics of the latter. A different pharmacogenomic profile among DMD patients could be one of the main factors determining the response to rimonabant. Future studies on a larger sample of patients are required to fully investigate this hypothesis.

Overactivity of the endocannabinoid system has been described in a large number of disorders including chronic inflammatory conditions involving both central and peripheral systems^51^. For example, in some metabolic dysfunctions caused by excessive inflammation and oxidative stress and characterized by elevated endocannabinoid tone and CB1 expression (i.e., obesity, high fat diet and alcohol-induced liver steatosis/fibrosis, high fat diet-induced β-cell dysfunction, doxorubicin-induced cardiomyopathy, high cholesterol-induced atherogenesis, etc.), CB1 inactivation reduces the infiltration of immune cells, fibrosis, and ROS production in several peripheral organs^52^. Thus, CB1 antagonism as an add-on therapy in DMD might also result in the sparing of steroidal anti-inflammatory drugs, which are responsible for serious side effects in children affected by this disease^53^. Additionally, recent studies (and our unpublished data) on the negative effects of CB1 activation on acetylcholine release from the neuromuscular junction^54–56^ suggest that CB1 antagonists may also effectively ameliorate muscle contraction. However, preliminary data from our laboratory on the lack of rimonabant effect on the isometric contraction of gastrocnemius muscles from 8 weeks old mdx mice, evoked by selective stimulation of the nerve in vivo, suggest that the beneficial effects of CB1 antagonism in the rotarod and weight test, reported here, may not be due to direct amelioration of muscle contraction performance.

In conclusion, we uncovered a potential vicious circle caused by excessive satellite cell proliferation and PAX7 overexpression, subsequent PAX7 binding to CB1, overexpression of this gene and CB1 overactivity, with further satellite cell proliferation and reduction of differentiation. We propose that the endocannabinoid system participates in the development of degenerative muscle disease, through effects on muscle differentiation, regeneration, and repair processes, and suggest that CB1 receptor may represent a potential target for the adjuvant therapy of muscle dystrophies.

## Methods

### Cell culture, reagents, and transfections

Primary human satellite cells were provided by Innoprot (Cat. no. P10976; Bizkaia-Spain) or Sciencell (Cat. no. 3510; Carlsbad, CA, USA). Satellite cells were propagated in a growth medium (GM) recommended by Innoprot (Skeletal Muscle Cell Medium, cat. no. P60124). Myotube differentiation was achieved after exposure of satellite cells to a skeletal muscle cell differentiation medium (DM) provided by Applied Stem Cell (Cat. no. ASE-5064; CA, USA).

Primary myoblasts were established from the muscle biopsy of DMD affected donors after they had signed informed consent forms and in accordance with the guidelines of the G. Gaslini Institute Ethical Committee. The myoblasts were grown in Full Aneural Medium [Dulbecco’s Modified Eagle Medium (DMEM) High glucose supplemented with 15% FBS, 20% Medium 199, 1% insulin, 1% glutamine, and 1% penicillin and streptomycin, FGF, EGF]. All the reagents were provided by Life Technologies (Milan, IT).

HEK293 (Human Embryonic Kidney-293; ATCC Number: CRL-11268) cells were propagated in Minimum Essential Media (MEM, cat. no. 31095-029 Invitrogen, Milan, IT) supplemented with 10% fetal bovine serum (FBS), 50 U/ml penicillin plus 50 µg/ml streptomycin, and 1% l-glutamine (Invitrogen, Milan, IT), in a humidified atmosphere of 95% air/5% CO_2_ at 37 °C.

For cell transfection, primary human satellite cells were plated in six multiwell culture dishes; the day after plating, cells were transfected with the esiRNA sequences targeting PAX7 (cat. no. EHU023151, Sigma-Aldrich, Milan, IT) using Lipofectamine RNAiMAX (cat. no. 13778150, ThermoFisher Scientific, Milan, IT) according to the manufacturer’s instructions.

HEK293 cells were seeded onto 24-well plastic plates at a 2 × 10^3^ cells/cm^2^ density. After plating, the cells were transfected on the next day with: (a) pGL3 promoter vector (Promega, IT; cat. no. E176A) with the portion of interest human or mouse CB1 gene cloned upstream the luciferase gene; (b) human PAX7 GFP-tagged plasmid (Origene, MD USA; cat. no. RG216836); (c) pCDNA3 (Life Technology, Milan, IT, cat. no. V79020); (d) a plasmid encoding for pSV-β-Galactosidase control vector (Promega, IT; cat. no. E1081) was used to normalize the signals and as positive control vector for monitoring transfection efficiency. The combination of plasmids was transfected into the cells by use of Lipofectamine LTX (Life Technology, MI IT) following the manufacture’s instruction.

The isolation of satellite cells from mouse skeletal muscle was performed using the gentleMACS^TM^ Dissociator (cat. no. 130-093-235) following the protocol of the skeletal muscle dissociation kit (cat. no. 130-098-305) and the satellite cell isolation kit, mouse (cat. no. 130-104-268). After isolation, cells were counted using an automatic cell counter (Scepter 2.0, Merk Millipore).

### Luciferase assay

After 48 h, the cells were harvested to detect the luciferase gene reporter activity by the use of the luciferase assay kit (cat. no. LUC1, Sigma-Aldrich MI, IT); whereas the β-galactosidase activity was detected with the β-galactosidase detection kit (cat. no. Gal-A, Sigma-Aldrich, Milan, IT). The signal intensity of β-Gal was measured at a microplate readers station (Tecan Geniuspro). Whereas, the signal intensity of firefly luciferase was detected on a ChemiDoc MP System station and reported as normalized with respect to the β-Gal.

### Bioinformatics and ChIP analysis

Putative PAX7 consensus sequences identification were identified using the match tool of Transfac. The ChIP assay was performed by the use of ChIP Kit (Sigma-Aldrich, Milan, IT; cat. no. CHP1) following the manufacturer’s instructions. The genomic DNA was sonicated, immunoprecipitated overnight at 4 °C with an antibody directed against PAX7 validated for ChIP (anti-PAX7 used at 2 µg/ml; Millipore, clone 2F12H4). Immunoprecipitated DNA was then analyzed by qPCR (40–45 cycles) using primers amplifying each of PAX7-rich regions identified (Supplemental Table 3). In addition, for each primer pair, non-immunoprecipitated DNA was used as input for normalization. All ChIP assays were performed in triplicate for at least three different biological preparations.

### Western blot and immunocytochemistry analysis

Satellite cells were washed two times in cold PBS (without Ca^2+^ and Mg^2+^, pH7.4) and lysed with lysis solution (150 mM NaCl, 1 mM EDTA, pH 7.4, 10 mM Tris-HCl, pH 8, 1% SDS, and 1% protease inhibitors). The same lysis solution was used to extract the protein from whole skeletal muscle tissues. Each animal was previously anesthetized, and once decapitated, the skeletal muscles were quickly dissected and washed twice in cold PBS. After the homogenization cell and/or tissue lysates were kept in orbital shaker incubator at 6 g 4 °C for 30 min and then centrifuged for 15 min at 13,000×*g* at 4 °C. The supernatants was transferred to clear tubes and quantified by DC Protein Assay (Bio-Rad, Milan, IT). Subsequently, the samples (70–80 μg of total protein) were boiled for 5 min in Laemmli SDS loading buffer and loaded on 8–10% SDS-polyacrylamide gel electrophoresis and then transferred to a PVDF membrane. Filters were incubated overnight at 4 °C with the following primary antibodies: (a) mouse anti-CB1 (1:500; cat. no. Y080037, Applied Biological Materials Inc.); mouse anti-PAX7 (1:500; SantaCruz CA USA, cat. no. sc-81648); rabbit anti PKC-pan (phospho Thr497; 1:500; Thermo Fisher Scientific; cat. no. GTX52316); rabbit anti PKC-pan total (1:500; Thermo Fisher Scientific; cat. no. GTX52352). An anti-α-tubulin antibody (1:5000; Sigma-Aldrich) was used to check for equal protein loading. Reactive bands were detected by chemiluminescence (ECL-plus; Bio-Rad). Reactive bands were detected by chemiluminescence (ECL-plus; Bio-Rad, Segrate, IT). The intensity of bands was analyzed on a ChemiDoc station with Quantity-one software (Bio-Rad, Milan, IT). Uncropped immunoblot image data. See Supplementary Figure 6 for uncropped images of key immunoblot data presented in this study. In immunocytochemistry analysis, primary human satellite cells were plated in growth media (GM) on glass coverslips treated overnight with poly-l-lysine (2 mg cm^−2^). The next day, GM was replaced with the differentiation media (DM) containing vehicle (DMSO) or rimonabant (1 µM). After 5 days of exposure to DM with/without rimonabant, the cells were harvested for qPCR analysis.

### Measurements of endocannabinoids and DAGs

Frozen muscle tissue samples were homogenized in chloroform/methanol/TRIS-HCl 50 mM, pH 7.4 (2:1:1, v/v), containing 5 pmol of [^2^H]_8_-AEA, 50 pmol of [^2^H]_5_-2-AG, [^2^H]_4_-PEA, and [^2^H]_2_-OEA as internal deuterated standards (purchased from Cayman Chemicals, Ann Arbor, MI, IT). The extract was purified by means of silica gel mini-columns, and the eluted fraction containing endocannabinoids and endocannabinoid-like molecules analyzed by means of liquid chromatography–atmospheric pressure chemical ionization–mass spectrometry (LC-APCI-MS). LC analysis was performed in the isocratic mode using a Discovery C18 column (15 cm × 4.6 mm, 5 µm) and methanol/water/acetic acid (85:15:0.1 by vol.) as mobile phase with a flow rate of 1 ml min^−1^. Analyses were carried out in the selected ion-monitoring mode using *m*/*z* values of 356 and 348 (molecular ions + 1 for deuterated and undeuterated AEA), 384.35, and 379.35 (molecular ions + 1 for deuterated and undeuterated 2-AG). AEA and 2-AG levels were therefore calculated on the basis of their area ratios with the internal deuterated standard signal areas. Lipid amounts expressed as pmol were then normalized per gram or milligram of wet tissue. To measure DAGs, satellite cells were dounce-homogenized and extracted with chloroform/methanol/Tris–HCl 50 mmol l^−1^ pH 7.5 (2:1:1, vol/vol) containing internal standard, the 1,2-heptadecanoin (Larodan AB, Malmo, Sweden). The lipid-containing organic phase was dried down, weighed, and pre-purified by open-bed chromatography on silica gel. Fractions were obtained by eluting the column with 99:1, 90:10, and 50:50 (v/v) chloroform/methanol. The 90:10 fraction was used for AEA and 2-AG quantification by LC-APCI-MS by using a Shimadzu high-performance liquid chromatography apparatus (LC-10ADVP) coupled to a Shimadzu (LCMS-2020) quadrupole mass spectrometry via a Shimadzu atmospheric pressure chemical ionization interface. LC analysis was performed in the isocratic mode using a Discovery C18 column (15 cm × 4.6 mm, 5 µm) and methanol/water/acetic acid (85:15:1 by vol.) as mobile phase with a flow rate of 1 ml min^−1^. The amounts of endocannabinoids in plasma, quantified by isotope dilution with the above mentioned deuterated standards, are expressed as pmol ml^−1^ of plasma volume or milligram of lipid extract weight. The 99:1 fraction was used for DAGs quantification by iquid chromatography-tandem mass spectrometry (LC-MS-MS) using an LC20AB coupled to a hybrid detector IT-TOF (Shimadzu Corporation, Kyoto, Japan) equipped with an ESI interface. DAGs were separated using a Discovery C18 column (15 cm × 2.1 mm, I.D. 5 μm; Supelco) and eluted with an isocratic flow of acetonitrile:2-propanol (85:15). LC20AB HPLC pumps were used to deliver solvent at a flow rate of 200 μl min^−1^. All molecular and fragment ions were sodiated and hence provide high-resolution values for [M + Na]^+^. We acquired full-scan MSn spectra of selected DAG precursor ions by multiple reaction monitoring (MRM), extracted the chromatograms of the high-resolution [M + Na]^+^ values and used the latter chromatograms for calibration and quantification. DAGs measured included 18:1-20:4 and 18:0-20:4 and the amounts of these species in cells were quantified using the standard, 1,2-heptadecanoin (17:0-17:0). DAG levels were measured by LC-MS-MS using an LC20AB coupled to a hybrid detector IT-TOF (Shimadzu Corporation, Kyoto, Japan) equipped with an ESI interface. *N* = 3–5 mice were used for these measurements. DAGs measured included 18:1-20:4, 18:0-20:4, and 16:0-20:4, and the amounts of these species in tissues were quantified using an external standard, 1,2-heptadecanoin (17:0-17:0, for which a calibration curve had been constructed in pilot experiments for the most abundant DAG species, 18:0-20:4, and considered valid also for the other DAG species), were expressed as pmols per mg of wet tissue weight. The calibration curve was also re-run occasionally, with no observed significant changes. All molecular and fragment ions were sodiated and hence provide high-resolution values for [M + Na]^+^. As most 20:4-containing DAG species are present in tissues in trace amounts; in some cases, we had to overload the column to be sure to detect these molecules. We acquired full-scan MSn spectra of selected DAG precursor ions by MRM, extracted the chromatograms of the high-resolution [M + Na]^+^ values, and used the latter chromatograms for calibration and quantification. The limit of detection (LOD) of 17:0-17:0 was 10 pmol in MS analysis.

### Muscle biopsies

All human samples were obtained after the patients had signed informed consent forms in accordance with the guidelines of the G. Gaslini Institute Ethics Committee, including experimental protocols for muscle biopsies. The muscle biopsy was obtained from femoral quadriceps muscle for diagnostic purposes from patients suspected for DMD. The diagnosis of DMD was also confirmed by genetic analysis.

### Animals care

The experimental protocol was evaluated and approved by the Institutional Animal Ethics Committee for the use of experimental animals and conformed to guidelines for the safe use and care of experimental animals in accordance with the Italian D.L. no. 116 of 27 January 1992 and associated guidelines in the European Communities Council (86/609/ECC and 2010/63/UE). For this study, control (C57BL/10ScSnJ) and dystrophic (C57BL/10ScSn-DMDmdx/J) mice of 5 weeks weighing ~20–25 g were purchased from Charles River Laboratories (MI, IT). All mice were housed in an individually ventilated cage system with a 12-h light–dark cycle and received standard mouse chow (Harlan Teklad) and water ad libitum.

### Drug treatment and reagents

Control (C57BL/10ScSnJ) or dystrophic (C57BL/10ScSn-DMDmdx/J) mice were intraperitoneally (IP) injected with: (a) vehicle (DMSO); (b) ACEA (2.5 mg Kg^−1^); or (c) rimonabant  (SR141716 0.5 mg Kg−1) three times per week for 2 weeks. DMSO and ACEA were from Sigma Aldrich. Rimonabant was from Cayman Chemical Company (USA).

### ELISA assays on plasma of control and dystrophic mice

Control and dystrophic mice treated with vehicle (DMSO) or rimonabant were previously anesthetized with halothane, subsequently the blood was removed by cardiac puncture and collected into EDTA-treated tubes. Blood cells were then removed by centrifugation for 10 min at 1000–2000×*g* using a refrigerated centrifuge. The resulting supernatant or plasma was further filtered in Amicon Ultra-0.5 ml Centrifugal Filters to concentrate the proteins with a molecular weight >10 kDa. The ELISA assay was performed by the use of either TNFα (cat. no. KHC3011) or IL-6 (cat. no. KMC0061) mouse ELISA Kits purchased from Thermo-Fisher scientific as per the manufacturer’s instruction.

### RNA-seq analysis

Hind limb muscles from normal wild-type C57/BL6 and C57Bl6 mdxdystrophic mice, used at the specified age of 8 weeks, were minced and digested in HBSS (Gibco) containing 2 mg ml^−1^ Collagenase A (Roche), 2.4 U ml^−1^ Dispase I (Roche), 10 ng ml^−1^ DNase I (Roche), 0.4 mM CaCl_2_, and 5 mM MgCl_2_ for 90 min at 37 °C. Cells were stained with primary antibodies CD31-PB (eBioscience), CD45-eFluor450 (eBioscience), Ter119-eFluor450 (eBioscience), F480-PE (eBioscience), CD11b-PC7, Sca-1-FITC (BD Pharmingen and a7integrin-APC (AbLab)) for 30 min on ice. Cells were finally washed and resuspended in HBSS containing 0.2% w/v BSA and 1% v/v penicillin–streptomycin. Flow cytometry analysis and cell sorting were performed on a DAKO-Cytomation MoFlo High Speed Sorter. Muscle satellite cells (SCs) were isolated as Ter119/CD45/CD31 negative (Lin−), a7-integrin-positive, and Sca-1-negative cells; FAP cells were isolated as Lin−, a7-integrin-negative, and Sca-1-positive cells. Macrophages (MPs) were isolated as Lin-positive, F480/CD11b-positive cells. Subsequently, for RNA-seq analysis, SCs, FAPs, and MPs freshly isolated by FACS from 16 wild-type C57/BL6 and six C57Bl6 mdx mice, 8-week old, were centrifuged and the pellet was resuspended in Trizol (Invitrogen) for total RNA extraction. About 100 ng µL^−1^ of total RNA was sent in duplicate to IGA for RNA-seq. RNA for the sequencing was processed using Illumina TruSeq Stranded Total RNA kit Ribo-Zero GOLD on Illumina Hiseq2500 platform. Mapping of more than 20 millions of reads for each sample to the mus musculus GRCm38.78 genome was performed using TopHat 2.0.9. Read count was performed with HTSeq-0.6.1p1. Mapped reads were analyzed with R-studio using DESeq2 to obtain normalized RPKM values and Heat-map with *Z*-score function. Sequencing data are available through SRA accession code SRP143532. For RT-PCR validation, 150 ng of total RNA, isolated with Trizol from SCs, FAPs, and MPs freshly isolated from wild-type C57/BL6 (*n* = 4) and three C57Bl6 mdx (*n* = 4) mice 8-week old, was retro-transcribed using the Taqman reverse transcription kit (Applied Biosystems). Real-time quantitative PCR was performed to analyze relative CB1, Daglα, and Magl gene expression levels using SYBR Green Master mix (Applied Biosystems) following manufacturer indications. The values were normalized to the housekeeping gene TBP for SCs and FAPs and to S16 for MPs.

### Rota-rod test

The rotarod test was performed in control and dystrophic mice immediately before the beginning of the pharmacological treatment (5 weeks) and at the indicated times following the treatment with vehicle, ACEA, or rimonabant. Briefly, the rotarod was settled with a start speed of 5 rpm and the mice were placed on the rotating rod for 30 s. Then, the rotarod was accelerated to 40 rpm in 240 s. The time (s) when mice dropped from the rod was recorded. The results were expressed as an average of two different trials and the interval time of each trial was 30 min.

### Weights test

To test the forelimb strength of dystrophic mice treated or not with ACEA and rimonabant, four weights of 20, 33, 46, and 59 g were used. Mice were handled by the base of the tail and were allowed to grip the first weight (20 g) and a hold of 3 s was the criterion. If the mouse dropped the weight in less than 3 s, we tried the same weight again for a maximum of three times. If the mouse held it for 3 s, then we tried it on the next heaviest weight. The mouse was assigned the maximum time/weight achieved. The final total score is calculated as the product of the number of links in the heaviest chain held for the full 3 s, multiplied by the time (s) it is held.

### Morphological analyses

For morphological analysis, gastrocnemius and quadriceps muscle were isolated from wild type, mdx mice injected with vehicle (DMSO) or rimonabant (SR141716, 0.5 mg Kg^−1^ IP). After dissection, the muscles were rapidly embedded in Sakura Tissue-Tek oct (Gentaur) and frozen in isopentane-cooled liquid nitrogen for cryosection or fixed in PFA and embedded in paraffin. Tissue necrosis was identified by morphological alterations of myofibers (i.e., hypercontraction) or loss of sarcolemma integrity and by the presence of cellular debris in the surrounding interstitial space. Regenerated myofibers were identified by the presence of central nuclei, and the diameter or CSA of fibers was morphometrically analyzed using KS300 image analysis software (Carl Zeiss) or Image-Quant software (Leica).

### Statistical analysis

We tested our datasets for normal distribution and chose an appropriate test accordingly using GraphPad Prism Version 7.0. Unpaired *t*-test was used to test two samples with equal variance. For more than two samples, we used one-way analysis of variance (ANOVA) followed by Bonferroni post hoc test. All error bars denote mean values ± SEM as indicated in every figure legend. The asterisk denote a *P* < 0.05 vs. the indicated experimental group.

## Electronic supplementary material

Supplementary Information

## Data Availability

Additional raw data that support the findings of this study are available from the corresponding authors upon reasonable request. RNAseq data are available through SRA accession code SRP143532.
