# Supplementary material for: Genetic and pharmacological regulation of the endocannabinoid CB1 receptor in Duchenne muscular dystrophy
Source: Nat Commun. 2018 Sep 27;9:3950. doi: 10.1038/s41467-018-06267-1 (PMC6160489; doi:10.1038/s41467-018-06267-1)
Supplement: Supplementary file 1 — Supplementary Information [file 41467_2018_6267_MOESM1_ESM.pdf]

SUPPLEMENTARY INFORMATION

# Genetic and pharmacological regulation of the endocannabinoid CB1 receptor in Duchenne Muscular Dystrophy

**Authors:**

Fabio A. Iannotti, Ester Pagano, Ombretta Guardiola, Simone Adinolfi, Valentina Saccone, Silvia Consalvi, Fabiana Piscitelli, Elisabetta Gazzerro, Giuseppe Busetto, Diego Carrella, Raffaele Capasso, Pier Lorenzo Puri, Gabriella Minchiotti and Vincenzo Di Marzo

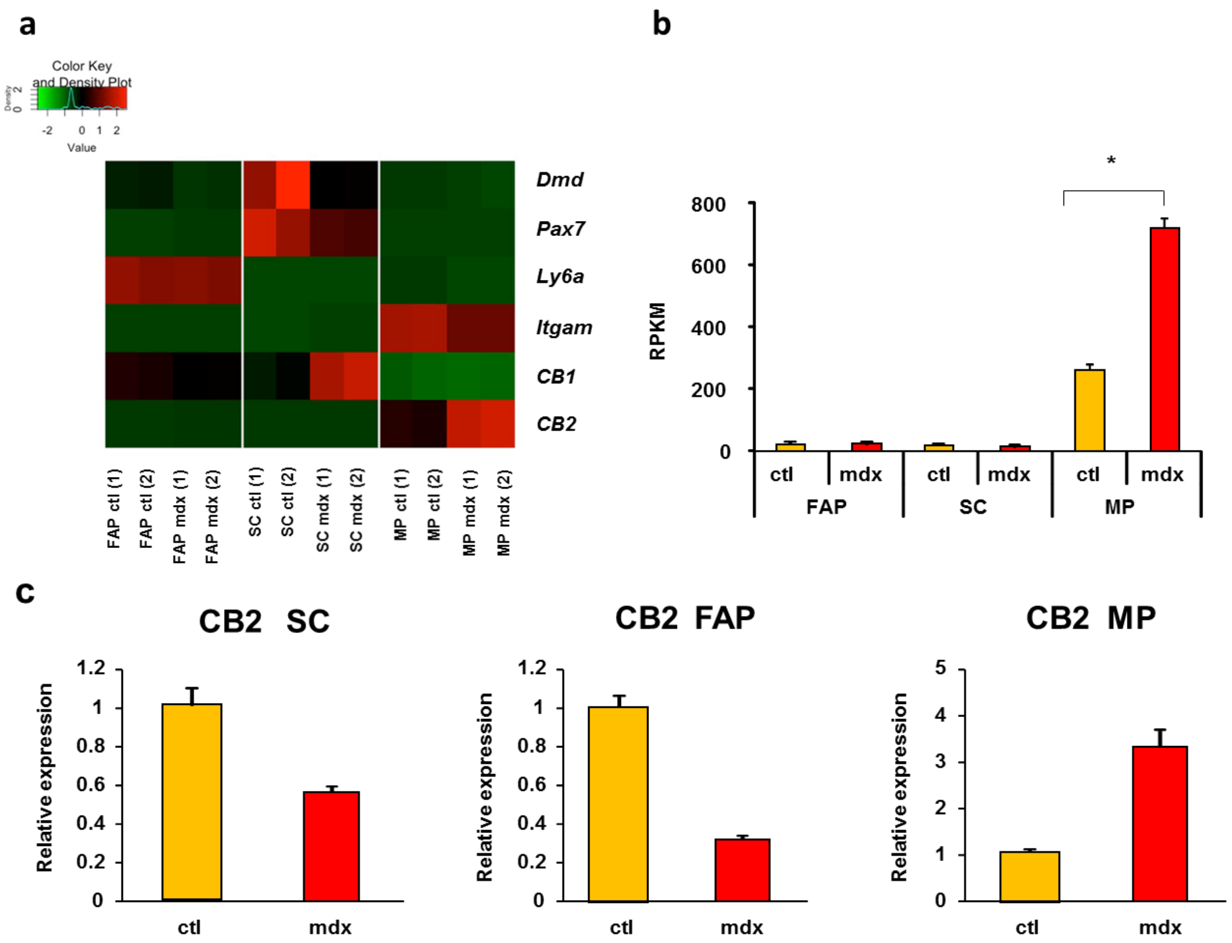


**Supplementary Figure 1** Expression of CB2 in skeletal muscle resident cells of mdx mice. **a** Heatmap representation of selected genes obtained from RNA-seq analysis in fibroadipogenic cells (FAP), satellite cells (SC) and macrophages (MP) isolated from 8-weeks old wt (n=4) and mdx (n=4) mice. Red, upregulated; green, downregulated **(B)** Bar Graph showing RPKM (Reads Per Kilobase of transcript per Million mapped reads) normalized values for the *Cnr2* gene obtained from RNA-seq analysis in FAP, SC and MP isolated from 8-weeks old wt and mdx mice. *P ≤ 0.05 vs control animals, determined by Student's t test. **(C)** qPCR validation data.

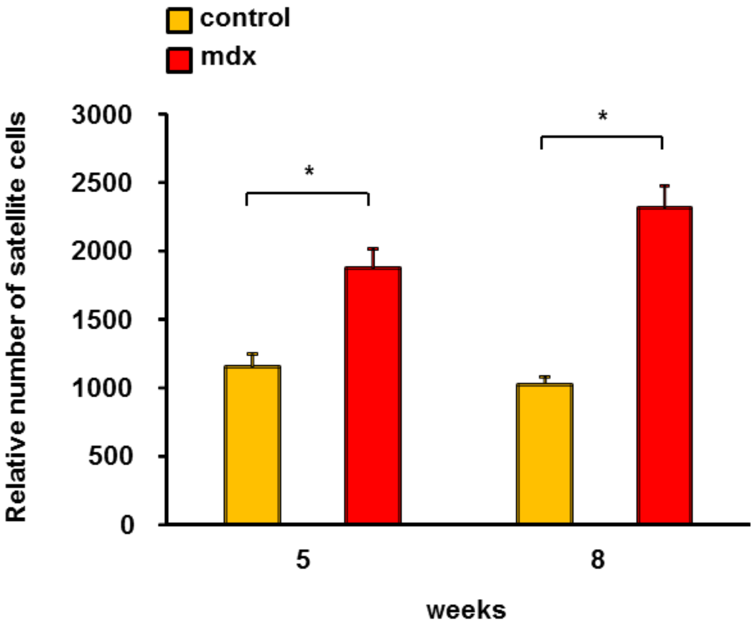


**Supplementary Figure 2** Relative number of satellite cells in control and mdx muscles. Bar graph showing the number of satellite cells found in the gastrocnemius muscles of control and mdx mice. Each bar is the mean ± SEM of four independent determinations. *P ≤ 0.05 vs control animals, determined by Student’s t test.

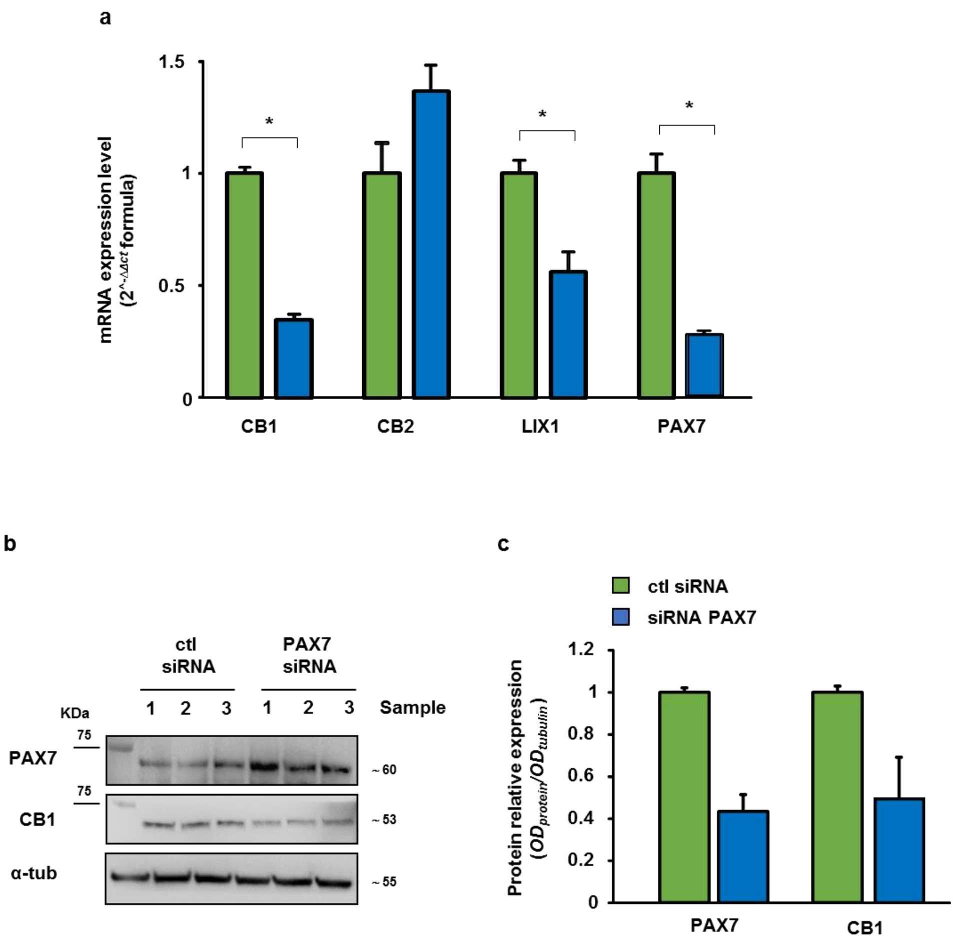


**Supplementary Figure 3** CB1 mRNA expression levels in PAX7-silenced satellite cells. **a** The quantification of CB1, CB2, LIX1 and PAX7 transcript levels was performed by quantitative real-time PCR. **b** Representative blots showing the chemiluminescent signal generated by the anti-CB1 or anti-PAX7 antibody in control (scramble) and PAX7-silenced satellite cells. **(C)** Quantification of CB1 and PAX7 protein expression levels normalized to α-tubulin. Each bar is the mean ± SEM of five separate determinations. *P ≤ 0.05 vs the control group, determined by Student's t test.

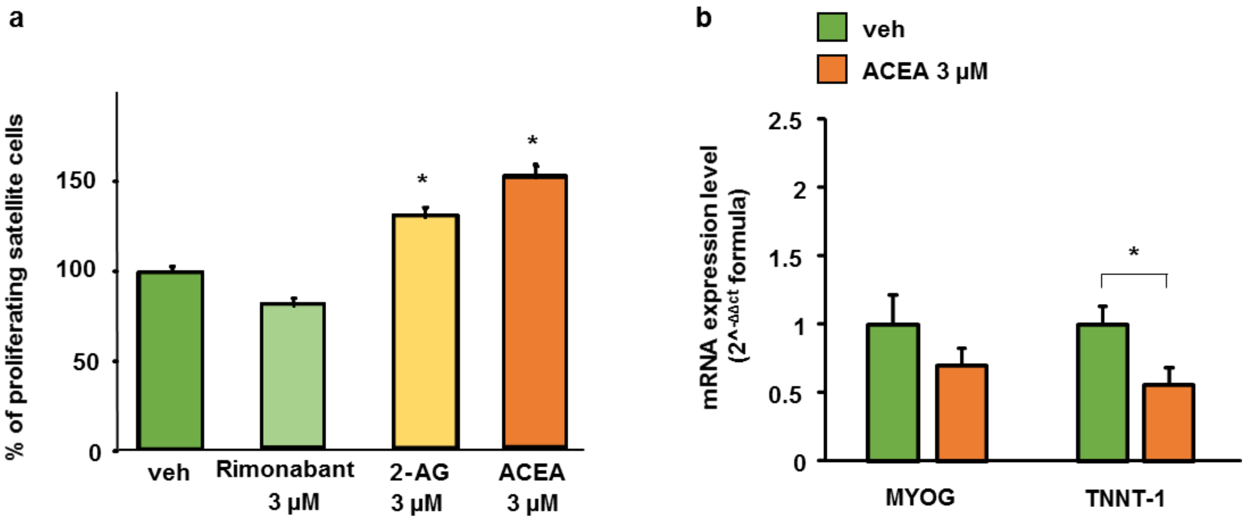


**Supplementary Figure 4** Pharmacological role of CB1 in human satellite cells **a** Effects of rimonabant (3 µM), 2-AG (3 µM) and ACEA ((3 µM) on satellite cell proliferation (n=4) assessed by incorporation of [$^3$H] thymidine. **b** Effect of ACEA on satellite cell differentiation. The quantification of myogenin (MYOG) and troponin-1 (TNNT-1) transcripts was performed by quantitative real-time PCR. Each bar is the mean ± S.E.M. of six separate determinations. *P ≤ 0.05 vs the control group, determined by Student's t test.

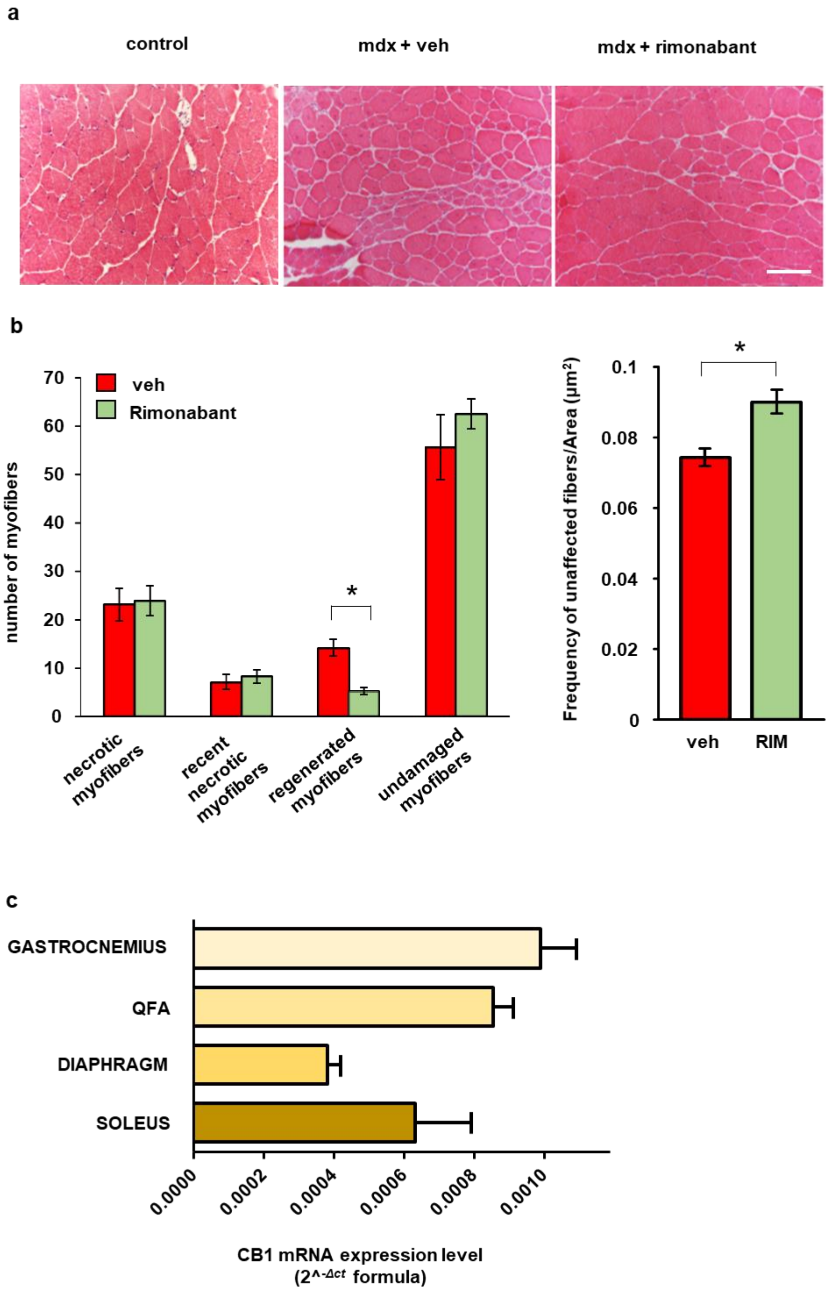


**Supplementary Figure 5** Histological analysis in mdx mice treated with rimonabant. **a** Representative photomicrographs of H&E-stained transverse sections at of quadriceps muscle isolated from wild-type (wt, n=6), mdx mice treated with vehicle (DMSO, n=6) or rimonabant 0.5 mg $Kg^{-1}$ (n=6) from week 5 to week 7 of age. Scale bars = 100 µm. **b** *Left* The bar graph indicates the number of necrotic, partly regenerated (recently necrotic), fully regenerated and undamaged myofibers in control (DMSO) and rimonabant-treated mdx mice. *Right* The bar graph indicates the number (frequency) of unaffected fiber/area ($µm^2$). **c** mRNA expression levels of CB1 mRNA in the gastrocnemius, soleus, diaphragm and quadriceps muscles of mdx mice (7 weeks) The quantification of transcripts for CB1 was performed by quantitative real-time PCR. Data are expressed as $2^{\wedge-\Delta ct}$ formula relative to S16, as described in Materials and Methods. Each bar is the mean ± S.E.M. of at least four separate determinations. *P ≤ 0.05 vs the indicated experimental group, determined by Student's t test.

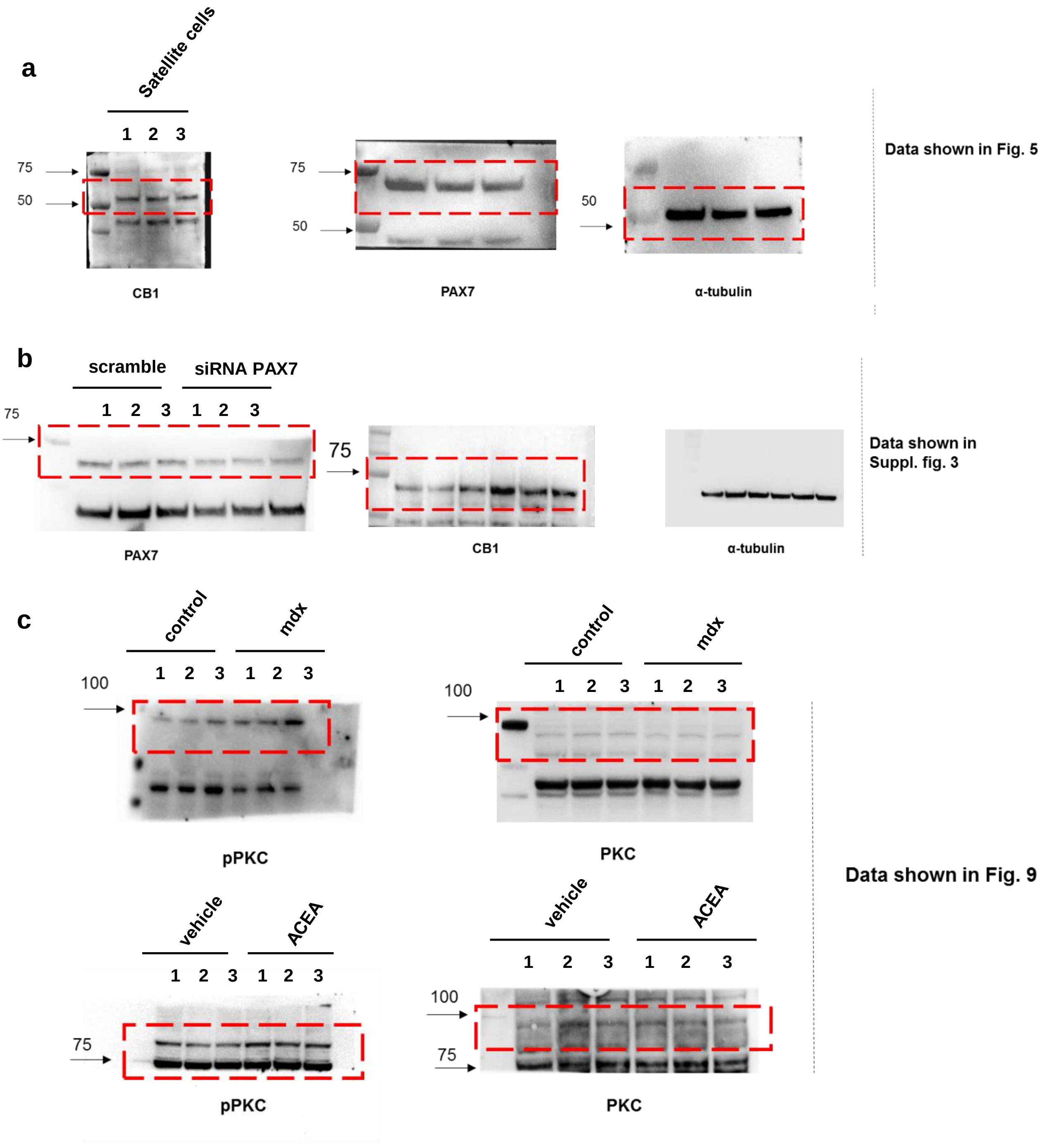


**Supplementary Figure 6:** Uncropped representative immunoblots shown from at least three independent experiments with anti-CB1, anti-PAX7, anti-PKC and anti pPKC antibodies obtained from satellite cells or skeletal muscle tissues from vehicle or ACEA treated mdx mice .

**Supplementary Table 1:** Endocannabinoid 2-AG levels in primary myoblasts isolated from DMD donors.

| Patient: | Vol of growth media (GM) | 2-AG levels normalized to vol. of GM (pmol ml$^{-1}$) | Amount of total lipid extract (mg) | 2-AG levels normalized to lipid extract (pmol mg$^{-1}$) |
|---|---|---|---|---|
| Healthy 1 | 12 | 1.44 | 1.08 | 15.01 |
| Healthy 2 | 12 | 0.78 | 1.43 | 5.99 |
| Healthy 3 | 12 | 0.99 | 1.10 | 7.54 |
| D1 | 12 | 1.09 | 1.20 | 9.75 |
| D2 | 12 | 1.20 | 1.24 | 9.86 |
| D5 | 12 | 1.44 | 1.12 | 16.29 |
| D6 | 12 | 1.00 | 0.96 | 12.96 |
| D8 | 12 | 1.06 | 1.31 | 10.64 |
| D9 | 12 | 1.05 | 1.17 | 10.87 |
| D10 | 12 | 1.97 | 1.30 | 17.91 |

**Supplementary Table 2:** Clinical indices of DMD patients

| Patient: | Sex: | Age: | Biopsy diagnosis: |
|---|---|---|---|
| Donor 1 (D1) | Male | 7 years 1 month | Exons 50-55 Deletion |
| Donor 2 (D2) | Male | 1 years 7 months | Exons 45-51 Duplication |
| Donor 3 (D3) | Male | 0 years 7 months | Exon 19 Puntiform mutation |
| Donor 4 (D4) | Male | 1 years 10 months | Exon 51 Deletion |
| Donor 5 (D5) | Male | 4 years 10 months | Exon 50 Deletion |
| Donor 6 (D6) | Male | 6 years 6 months | Exon 45 Deletion |
| Donor 7 (D7) | Male | 3 years 4 months | Exon 45 Deletion |
| Donor 8 (D8) | Male | 3 years 11 months | Exon 25 Stop mutation |
| Donor 9 (D9) | Male | 4 years 9 months | Exons 19-24 Deletion |

**Supplementary Table 3:** List of primers used in qPCR analysis

| Gene | FORWARD Sequence (5'->3') | REVERSE Sequence (5'->3') |
|---|---|---|
| Hum CB1 | TCTGTTCATCGTGTATGC | CTTGGCTAACCTAATGTC |
| Murine CB1 | GGGCACCTTCACGGTTCTG | GTGGAAGTCAACAAAGCTGTAGA |
| Murine troponin-T (Tnnt-1) | CTGTGGTGCCTCCTTTGATTC | TGCGGTCTTTTAGTGCAATGAG |
| Murine myogenin (Myog) | ATGGAGCTGTATGAGACATCCC | TTACACACCTTACATGCCCAC |
| Hum troponin-T (TNNT-1) | TGATCCCGCCAAAGATCC | TCTTCCGCTGCTCGAAATGTA |
| Hum myogenin (MYOG) | GGGGAAAACTACCTGCCTGTC | AGGCGCTCGATGTACTGGAT |
| Murine MyHC | AGGCGGACCTACTGTAAC | AGAGATGGAGAAGATGTGG |
| Murine neoMyHC | GTCACGCAATGCAGAAGAGA | CAGGTCCTTCACCGTCTGTT |
| Murine TNFα | GCGGCCACAGAAAACACTC | CTCCCAACTGGTCAAGGCATC |
| Murine IL6 receptor | AGCGACACTGGGGACTTA | ACAGCCTTCGTGGTTGGAG |
| Hum PAX7 | AGCACTGTGCCCTCAGTGAGTT | TCCGACTGCGTCGCTGCTTG |
| Murine PAX7 | CTCAGTGAGTTCGATTAGCCG | AGACGGTTCCCTTTGTCGC |
| Murine TGFβ | CTTCAATACGTCAGACATTCGGG | GTAACGCCAGGAATTGTTGCTA |
| Reg. 2 | TCAACCCAAGGCCACTTAGG | GGTTCAGTCCTCAGCTCCAG |
| Reg. 3 | AGAGTGTCTGGCCTTCCTCA | CTTGCTCCCGTATTCCAAGA |
| Reg. 4 | ACCTTACGACTCGACTGGAC | CGCAGGAATTAGGGGACATG |
| Hum S16 | TCGGACGCAAGAAGACAGCGA | AGCGTGCGCGGCTCAATCAT |
| murine S16 | CTGGAGCCTGTTTTGCTTCTG | TGAGATGGACTGTCGGATGG |
| TBP | CTGGAATTGTACCGCAGCTT | TCCTGTGCACACCATTTTTC |
